# Biogeographical Divergence of the Flora of Yunnan, Southwestern China Initiated by the Uplift of Himalaya and Extrusion of Indochina Block

**DOI:** 10.1371/journal.pone.0045601

**Published:** 2012-09-24

**Authors:** Zhu Hua

**Affiliations:** Key Laboratory of Tropical Forest Ecology, Xishuangbanna Tropical Botanical Garden, Chinese Academy of Sciences, Kunming, Yunnan, People’s Republic of China; Centro de Investigación y de Estudios Avanzados, Mexico

## Abstract

The floral composition of Yunnan is conspicuously linked to the biogeographical history of this extremely species-rich province in southwestern China. The floristic compositions of three representative regions in Yunnan were compared to reveal their variation with geography. From southern Yunnan, 4150 native species (including subspecies and varieties) from 1240 genera and 183 families of seed plants were recognized. From central Yunnan 3389 native species from 1095 genera and 167 families of seed plants were recognized. From northwestern Yunnan 6807 native species from 1296 genera and 166 families of seed plants were recognized. Although these three floras across Yunnan are similar in familial composition, similarities between the floras of southern and northwestern Yunnan are low at the generic and specific levels. The flora of northwestern Yunnan is dominated by families and genera with cosmopolitan and north temperate distributions, while the flora of southern Yunnan is dominated by tropical families and genera. Northwestern Yunnan is composed largely of temperate genera, of which the highest proportion has a north temperate distribution. In contrast, southern Yunnan has mainly tropical genera, of which most have a tropical Asian distribution. The flora of central Yunnan is a combination of southern and northwestern Yunnan. These three floras might be derived from a common Tertiary tropical or subtropical East Asian flora, but the geological history of each region has influenced its flora, and they have remained divergent since the late Tertiary. The flora of northwestern Yunnan has evolved with the uplift of the Himalayas and by gradual proliferation of mainly cosmopolitan and north temperate floristic elements, while the flora of southern Yunnan has evolved with extrusion of the Indochina block and the influence of mainly tropical Asian elements.

## Introduction

The “Eastern Asiatic floristic region” was delineated by Takhtajan [1] in his floristic regionalization of the world. The region is considered to be one of the major centres of development of higher plants, as it is especially rich in gymnosperms and primitive angiosperms [2]. Yunnan province of south-western China is the core area of the west wing of the “Eastern Asiatic floristic region” with extremely rich biodiversity, and is among the hotspots for conservation priorities in the world [3]. The region is also important for understanding historical biogeography due to its location in a transitional zone between tropical south-east Asia and temperate east Asia, and its position in geological history at a sutural zone between Gondwana and Laurasia [4], [5].

Yunnan is a mountainous region with an extremely diverse topography (from 76.4 m at the lowest valley in the southeast to 6740 m at the highest mountain summit in the northwest). Due to its diverse topography and climate, as well as its unique geological history, Yunnan is extremely rich in species and vegetation types, and the landscape varies vastly from tropical rain forests in southern Yunnan, to Taiga-like cold temperate coniferous forest in northwestern Yunnan.

The plant geography of Yunnan was primarily studied by Li and Walker [6]. There are many floristic works on local areas and nature reserves in Yunnan, with fewer large area and regional scale studies, but exceptions are the large Hengduan Mountains [7], [8], [9], southern Yunnan [10], [11], southwestern Yunnan [12], [13], and central Yunnan [14]. Southern Yunnan, with a tropical monsoon climate and lower mountain-basin topography, has a tropical flora of Malaysian affinity. Central Yunnan, with a subtropical climate and middle mountain-valley topography, has close affinities to the subtropical flora of East Asia. Northwestern Yunnan, with a temperate climate and alpine-deep valley topography, has a temperate Himalayan flora. These patterns in the flora and vegetation of Yunnan are of extreme interest to botanists. In the present study, we select three representative regions with different altitudes across this extremely biodiverse region of China, southern, central and northwestern Yunnan, to study their floristic compositions and variation with geography, and their evolution with geological history, as well as biogeographical affinities.

### General Geography

**Figure 1:**
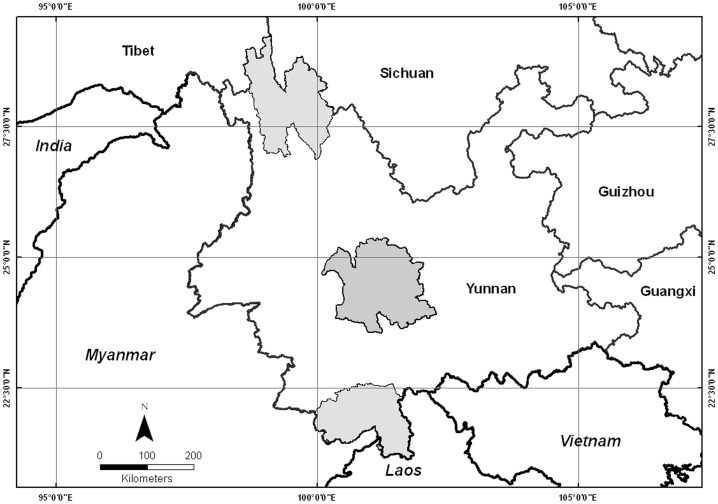
Map showing the study areas: Northwestern, Central and Southern Yunnan.

Yunnan is in southwestern China between 21°09′ and 29°15′ N, 97°32′ and 106°12′ E (Figure 1) and occupies an area of 394,100 km^2^. It has a mountainous topography with the mountain ridges generally running in a north-south direction, decreasing in elevation southward. Yunnan is extremely diverse in habitat and topography. The general climatic pattern consists of tropical wet climates in the southern lowlands (annual mean temperature 19–22°C), tropical dry climates in deep valleys below 1000 m alt. (20–24°C) due to the foehn effect, subtropical climates on the central plateau (14–18°C), and temperate to cold temperate climates in the northern high mountains (5–14°C). The climate changes conspicuously with altitude. Yunnan is therefore a region with tropical areas as the horizontal base [15].

Yunnan supports an extremely rich biodiversity and various vegetation types. A total of 13,245 species in 2,137 genera and 222 families of native seed plants are recognized from Yunnan [16], contributing to 50% of the total seed plant species in China. Twelve vegetation types including 169 formations were recognized in the vegetation classification of Yunnan [17], including tropical rain forest, subtropical evergreen broad-leaved forest, warm-temperate deciduous broad-leaved forest, temperate coniferous and broad-leaved mixed forest, cold temperate coniferous forest, alpine shrubs and meadows, as well as thorny shrubs and savanna in dry-hot valleys [18]. Southern Yunnan is generally covered by tropical rain forest at its lowlands [19]. Most of central Yunnan is between 1300–2200 m, and is covered by subtropical evergreen broad-leaved forest and secondary *Pinus yunnanensis* forest. Northern Yunnan, with an elevation above 1900 m, is covered mainly by temperate sclerophyllous oak forest and cold-temperate coniferous forest [20]. The distribution of vegetation in Yunnan corresponds more with elevation than latitude [10].

## Materials and Methods

This study focuses on three representative regional floras from southern, central and northwestern Yunnan. Southern Yunnan (Xishuangbanna administrative region) lies between 21°09′ and 22°36′ N, 99°58′ and 101°50′E and has an area of 19690 km^2^ with altitude varying from 480 m at the bottom of the lowest valley (Mekong River) to 2430 m at the highest mountain top. Central Yunnan, here including 7 counties, lies between 23°53′ and 25°11′ N, 100°32′ and 101°58 ′E. It includes the core area of Wuliang and Ailao Mountains, and has an area of 25,424 km^2^. Its altitude varies from 422 m at the lowest valley in the south (Red River) to 3156.9 m at the highest mountain top. Northwestern Yunnan, here including three counties, lies between 27°10′ and 28°27′ N, 98°53′ and 99°42′ E. It has an area of 23870 km^2^ with altitude varying from 1900 m at the lowest valley to 6740 m at the highest mountain summit (Figure 1).

Complete lists of the total native seed plants of southern, central and northwestern Yunnan were based on the recently completed Flora of Yunnan [16], the data base of seed plants from KUN (herbarium of Kunming Institute of Botany, Chinese Academy of Sciences), and floristic inventories [21], [22], [23]. Circumscription of families and species followed the nomenclature of w^3^TROPICOS (http://mobot.mobot.org/W3T/Search/vast.html).

Based on these plant lists, floristic and geographical attributes of the floras of the three areas were analyzed. Patterns of seed plant distributions of these floras were quantified at the generic level based on Wu’s documentation [24] and at the family level following Wu et al. [25]. Comparisons of both floristic composition and geographical elements were made to assess floristic similarities and variation, as well as to determine biogeographical affinities between the three areas.

## Results

### Floristic Composition

A total of 4150 native species including subspecies and varieties from 1240 genera and 183 families of seed plants were recognized from southern Yunnan. Families with highest species richness include Orchidaceae (377 species and taxa under species), Fabaceae (261), Rubiaceae (201), Poaceae (189), Euphorbiaceae (148), Lamiaceae (139), Asteraceae (137), Lauraceae (105), Urticaceae (84), Zingiberaceae (84) and Moraceae (83)).

A total of 3389 native species including subspecies and varieties from 1095 genera and 167 families of seed plants were recognized from central Yunnan. Families with the highest species richness include Asteraceae (202 species and taxa under species), Fabaceae (187), Poaceae (183), Lamiaceae (132), Orchidaceae (121), Rubiaceae (121), Rosaceae (119) and Ericaceae (87).

A total of 6807 native species including subspecies and varieties from 1296 genera and 166 families of seed plants were recognized from northwestern Yunnan. Families with the highest species richness include Asteraceae (518 species), Poaceae (395), Rosaceae (358), Orchidaceae (323), Fabaceae (299), Ericaceae (284), Ranunculaceae (238), Lamiaceae (220), Apiaceae (198), Orobanchaceae (181), Cyperaceae (176), Primulaceae (146), Gentianaceae (145), Saxifragaceae (124), Salicaceae (121), Liliaceae (113), Rubiaceae (111) and Caryophyllaceae (110).

The dominant families from these three floras are summarised in Table 1.

**Table 1:** Dominant families ranking by species richness of these three compared floras.

Flora of southern Yunnan			Flora of central Yunnan			Flora of northwestern Yunnan		
Family	No. sp.	Sp.%	Family	No. sp.	Sp.%	Family	No. sp.	Sp.%
Orchidaceae	377	9.08	Asteraceae	202	5.99	Asteraceae	518	7.61
Fabaceae	261	6.29	Fabaceae	187	5.52	Poaceae	395	5.80
Rubiaceae	201	4.84	Poaceae	183	5.40	Rosaceae	358	5.26
Poaceae	189	4.55	Lamiaceae	132	3.89	Orchidaceae	323	4.75
Euphorbiaceae	148	3.57	Orchidaceae	121	3.57	Fabaceae	299	4.39
Lamiaceae	139	3.35	Rubiaceae	121	3.57	Ericaceae	284	4.17
Asteraceae	137	3.30	Rosaceae	119	3.51	Ranunculaceae	238	3.50
Lauraceae	105	2.53	Ericaceae	87	2.57	Lamiaceae	220	3.23
Urticaceae	84	2.02	Euphorbiaceae	69	2.04	Apiaceae	198	2.91
**Zingiberaceae**	84	2.02	Urticaceae	65	1.92	Cyperaceae	176	2.59
Moraceae	83	2.00	Lauraceae	63	1.86	Orobanchaceae	181	2.66
Acanthaceae	77	1.86	Liliaceae	61	1.80	Primulaceae	146	2.14
Asclepiadaceae	66	1.59	Orobanchaceae	61	1.80	**Gentianaceae**	145	2.13
Cyperaceae	63	1.52	Moraceae	59	1.74	**Saxifragaceae**	124	1.82
**Cucurbitaceae**	60	1.45	Fagaceae	57	1.68	**Salicaceae**	121	1.78
Fagaceae	60	1.45	Ranunculaceae	52	1.53	Liliaceae	113	1.66
Rosaceae	59	1.42	Primulaceae	49	1.45	Rubiaceae	111	1.63
**Annonaceae**	57	1.37	Polygonaceae	47	1.39	**Caryophyllaceae**	110	1.62
**Apocynaceae**	56	1.35	**Theaceae**	45	1.33	Polygonaceae	99	1.45
Vitaceae	56	1.35	Araliaceae	44	1.30	Urticaceae	99	1.45
Orobanchaceae	55	1.33	Asclepiadaceae	44	1.30	**Cruciferae**	94	1.38
Araceae	47	1.13	Cyperaceae	42	1.24	Caprifoliaceae	85	1.25
Rutaceae	46	1.11	Gesneriaceae	41	1.21	**Papaveraceae**	83	1.22
**Convolvulaceae**	42	1.01	Rutaceae	40	1.18	**Campanulaceae**	78	1.15
Gesneriaceae	42	1.01	Acanthaceae	38	1.12	Araliaceae	71	1.04
**Arecaceae**	41	0.99	Caprifoliaceae	36	1.06	**Berberidaceae**	69	1.01
**Sterculiaceae**	40	0.96	Apiaceae	36	1.06	Lauraceae	65	0.95
Malvaceae	38	0.92	Celastraceae	35	1.03	**Crassulaceae**	64	0.94
Polygonaceae	38	0.92	Gentianaceae	33	0.97	Gesneriaceae	61	0.90
Celastraceae	37	0.89	Vitaceae	33	0.97	**Sapindaceae**	56	0.82

The families showed by bold letter are the dominant families, which are only in one of the three floras respectively.

Genera with the highest species richness in southern Yunnan include *Ficus* (65 species), *Dendrobium* (47), *Bulbophyllum* (40), *Polygonum* (34) and *Litsea* (30). The dominant genera in central Yunnan include *Ficus* (49), *Rubus* (38), *Polygonum* (37), *Rhododendron* (33) and *Primula* (30); while the dominant genera in northwestern Yunnan are *Rhododendron* (183), *Pedicularis* (119), *Salix* (105), *Carex* (93), *Primula* (93), *Gentiana* (90), *Saxifraga* (86), *Saussurea* (71), *Polygonum* (65), *Rubus* (64), *Corydalis* (59), *Aconitum* (56), *Berberis* (56), *Acer* (54), *Astragalus* (52) and *Ligularia* (50) (Table 2).

**Table 2:** Dominant genera ranking by species richness of these three compared floras.

Genera in southern Yunnan	No. of species	Genera in central Yunnan	No. of species	Genera in northwestern Yunnan	No. of species
*Ficus*	65	*Ficus*	49	*Rhododendron*	183
*Dendrobium*	47	*Rubus*	38	*Pedicularis*	119
*Bulbophyllum*	40	*Polygonum*	37	*Salix*	105
*Polygonum*	34	*Rhododendron*	33	*Carex*	93
*Litsea*	30	*Primula*	30	*Primula*	93
*Dioscorea*	28	*Clematis*	25	*Gentiana*	90
*Syzygium*	27	*Lithocarpus*	25	*Saxifraga*	86
*Calamus*	25	*Ilex*	23	*Saussurea*	71
*Piper*	25	*Euonymus*	20	*Polygonum*	65
*Begonia*	24	*Litsea*	20	*Rubus*	64
*Eria*	22	*Symplocos*	20	*Corydalis*	59
*Tetrastigma*	22	*Vaccinium*	20	*Aconitum*	56
*Desmodium*	21	*Smilax*	19	*Berberis*	56
*Elatostema*	21	*Camellia*	18	*Acer*	54
*Lithocarpus*	21	*Desmodium*	18	*Astragalus*	52
*Strobilanthes*	21	*Lysimachia*	18	*Ligularia*	50
*Castanopsis*	20	*Viola*	18	*Ilex*	46
*Lasianthus*	20	*Elsholtzia*	17	*Juncus*	43
*Smilax*	20	*Impatiens*	17	*Poa*	41
*Millettia*	19	*Pilea*	17	*Cotoneaster*	39
*Amomum*	18	*Carex*	16	*Delphinium*	39
*Clerodendrum*	18	*Elatostema*	16	*Silene*	39
*Elaeocarpus*	18	*Piper*	16	*Potentilla*	38
*Habenaria*	18	*Schefflera*	16	*Arenaria*	37
*Ophiorrhiza*	18	*Tetrastigma*	16	*Arisaema*	37
*Rubus*	18	*Crotalaria*	15	*Aster*	36
*Crotalaria*	17	*Dioscorea*	15	*Euonymus*	36
*Oberonia*	17	*Eurya*	15	*Sorbus*	36
*Vernonia*	17	*Gentiana*	15	*Clematis*	35
*Glochidion*	16	*Begonia*	14	*Indigofera*	35

### Geographical Elements

#### Geographical elements at family level

Twelve distribution types of family are recognized from northwestern, central, and southern Yunnan (Table 3).

**Table 3:** Geographical elements of seed plants at the family level of these three compared floras.

Geographical elementsat family level	Flora of southern Yunnan				Flora of central Yunnan				Flora of northwestern Yunnan				
	No. of family		%*		No. of family		%		No. of family		%		
Cosmopolitan		47		25.68		47		28.14		50		30.12	
Pantropic		77		42.08		67		40.12		57		34.34	
Tropical Asia and Tropical America disjunct		11		6.01		10		5.99		11		6.63	
Old World Tropic		3		1.64		2		1.20		2		1.20	
Tropical Asia to Tropical Australia		5		2.73		3		1.80		3		1.81	
Tropical Asia to Tropical Africa		1		0.55		1		0.60		0		0.00	
Tropical Asia		9		4.92		4		2.40		2		1.20	
North Temperate		19		10.38		21		12.57		24		14.46	
East Asia and North America disjunct		7		3.83		7		4.19		9		5.42	
Old World Temperate		0		0.00		0		0.00		1		0.60	
East Asia		3		1.64		4		2.40		6		3.61	
Endemic to China		1		0.55		1		0.60		1		0.60	
Total		183		100.00		167		100.00		166		100.00	

* The number of family in each geographical element/the number of family of all geographical elements.

In the flora of southern Yunnan, there are 106 (57.92%) families with tropical distributions. Those with pantropic distributions make up 73.9% of the tropically distributed families, and 42.08% of the total number of families, such as Acanthaceae, Anacardiaceae, Annonaceae, Apocynaceae, Araceae, Arecaceae, Burseraceae, Clusiaceae, Myristicaceae, Sapotaceae and Icacinaceae. The remainder have old world tropic distributions (including Pittosporaceae, Pandanaceae and Musaceae), tropical Asian and tropical American disjunct distributions (including Araliaceae, Elaeocarpaceae, Gesneriaceae, Staphyleaceae and Styracaceae), and tropical Asian distributions (such as Crypteroniaceae and Sabiaceae). Cosmopolitan families make up 25.68% of the total number of families in southern Yunnan, such as Asteraceae, Poaceae, Rosaceae, Fabaceae etc. Families with mainly temperate distributions contribute 16.39% to the total flora, including those with north temperate distributions (such as Caprifoliaceae, Betulaceae, Buxaceae and Salicaceae), east Asia and north America disjunct distributions (Magnoliaceae, Nyssaceae and Saururaceae), and east Asian distributions (Actinidiaceae, Cephalotaxaceae and Stachyuraceae).

In the flora of central Yunnan, there are 87 (52.10%) families with tropical distributions, including 67 (40.12%) families with pantropic distributions. Cosmopolitan families contribute to 28.14% of the total number of families. Families with mainly temperate distributions make up 19.76% of the total flora, including 21 families of north temperate distribution and seven families of east Asia and north America disjunct distributions.

In the flora of northwestern Yunnan, 45.18% of families have tropical distributions, of which 57 (34.34% of the total number of families) have pantropic distributions. The proportion of families with cosmopolitan distributions is 30.12%. There are 24 families (14.46%) with north temperate distributions, and 5.42% have east Asia and north America disjunct distributions.

#### Geographical elements at the generic level

Distributions of the seed plants at the generic level are summarized in Table 4.

**Table 4:** Geographical elements of seed plants at the generic level of these three compared floras.

Geographical elementsat generic level	Flora of southern Yunnan		Flora of central Yunnan		Flora of northwestern Yunnan	
	No. of genus	%*	No. of genus	%	No. of genus	%
Cosmopolitan	59	4.76	61	5.57	76	5.86
Pantropic	254	20.48	204	18.63	192	14.80
Tropical Asia and Tropical America disjunct	31	2.50	35	3.20	30	2.31
Old World Tropic	123	9.92	96	8.77	81	6.25
Tropical Asia to Tropical Australia	132	10.65	82	7.49	64	4.93
Tropical Asia to Tropical Africa	71	5.73	57	5.21	46	3.55
Tropical Asia	344	27.74	187	17.08	142	10.95
North Temperate	68	5.48	138	12.60	209	16.11
East Asia and North America disjunct	32	2.58	43	3.93	71	5.47
Old World Temperate	29	2.34	45	4.11	99	7.63
Temperate Asia	5	0.40	6	0.55	18	1.39
Mediterranean, W Asia to C Asia	3	0.24	5	0.46	16	1.23
Center Asia	2	0.16	2	0.18	14	1.08
East Asia	72	5.81	115	10.50	185	14.26
Endemic to China	15	1.21	19	1.74	53	4.09
Total no. of genera	1240	100.00	1095	100.00	1296	100.00

* The number of genera in each geographical element/the number of genera of all geographical elements.

In southern Yunnan, genera with tropical distributions (Table 4, types 2–7) comprise 77.02%, and those with tropical Asian distributions (e.g., *Alphonsea, Amoora, Pterospermum, Mitrephora, Mycetia, Aganosma, Chukrasia, Crypteronia,* and *Knema*) are most common, contributing to 27.74% of the flora. Genera with pantropic distributions, such as *Gnetum, Beilschmiedia, Cryptocarya, Capparis, Piper, Dioscorea, Uncaria* make up 20.48% of the flora. Genera with old world tropical distributions, such as *Thunbergia, Dracaena, Pandanus, Ventilago, Stephania, Fissistigma, Polyalthia,* and *Uvaria,* make up 9.92%, and 10.65% of genera have Asia to tropical Australia distributions, including *Ailanthus, Hoya, Argyreia, Dillenia, Lagerstroemia, Murraya* and *Toona.* Genera with temperate distributions (Table 4, types 8–14) contribute to 17.02%, including genera with north temperate distributions (e.g., *Artemisia, Carpinus, Betula, Salix,* and *Sorbus*), east Asia and north America disjunct distributions (e.g., *Schizandra, Photinia, Nyssa, Magnolia, Mahonia* and *Castanopsis*), old world temperate distributions (e.g., *Ajuga, Elsholtzia, Ligustrum* and *Paris*), and east Asian distributions (*Actinidia, Belamcandia, Aspidistra, Cephalotaxus* and *Pterocarya*). Only 15 genera are endemic to China, including *Biondia*, *Camptothec, Craspedolobium, Cyphotheca*, *Dichotomanthe, Eleutharrhena, Nouelia*, *Paramomum*, *Styrophyton, Tapiscia* and *Thyrocarpus*.

In the flora of central Yunnan, genera with tropical distributions (Type 2–7) make up 60.37% of the total genera, of which genera with pantropic distributions are most common (18.63%), followed by those with tropical Asia distributions (17.08%). Genera with temperate distributions (Type 8–14) in all make up 32.33% of the total genera, and those with north temperate distributions make up 12.60% of the total genera, followed by those with east Asia distributions, which make up 10.50% of the total genera.

In northwestern Yunnan, there are 612 (47.19%) genera with temperate distributions (Type 8–14). Among them, genera with northern temperate distributions (e.g., *Abies, Pinus, Carpinus, Betula, Salix* and *Populus*), contribute to 16.11% of the total genera. Genera with east Asian distributions (e.g., *Actinidia, Ainsliaea, Aucuba, Aspidistra, Cephalotaxus, Fargesia, Helwingia, Hovenia* and *Yushania*) contribute to 14.26% of the total genera. Genera with tropical distributions (Type 2–7) make up 42.87% of the genera, and most have pantropic (14.80%) (e.g., *Adenostemma, Ardisia, Bauhinia, Buxus, Diospyros, Smilax* and *Vitex*) or tropical Asian (10.95%) distributions (e.g., *Actinodaphne, Agapetes, Camellia, Cipadessa, Daphniphyllum, Engelhardtia* and *Exbucklandia*). There are 53 genera that are endemic or approximately endemic to China, including *Davidia, Dipelta, Kingdonia, Musella, Ostryopsis, Taiwania* etc.

### Biogeographical Divergence of the Flora of Yunnan

The floristic similarity between these regional floras of Yunnan is more than 88% at the family level and more than 55.89% at the generic level, and lowest at the species level (26.72%–48.45%) (Table 5). Central Yunnan shares a nearly equal proportion of flora with both southern and northwestern Yunnan. The family Theaceae is particularly species rich in central Yunnan, but otherwise the dominant families of central Yunnan are also common to southern or northwestern Yunnan.

**Table 5:** Comparison of floristic similarities at the family, generic and specific levels between these floras of southern, centre and north-western Yunnan.

Compared flora	Southern Yunnan	Central Yunnan	Northwestern Yunnan
	Shared/Similarity coefficient (%)*	Shared/Similarity coefficient (%)	Shared/Similarity coefficient (%)
Similarity coefficients at family level			
Southern Yunnan	100/100		
Central Yunnan	157/94.01%	100/100	
Northwestern Yunnan	147/88.55	151/90.96	100/100
Similarity coefficients at generic level			
Southern Yunnan	100/100		
Central Yunnan	808/73.63	100/100	
Northwestern Yunnan	693/55.89	833/76.00	100/100
Similarity coefficients at specific level			
Southern Yunnan	100/100		
Central Yunnan	1527/45.06		
Northwestern Yunnan	1109/26.72	1642/48.45	

* Similarity coefficient between A and B = the number of taxa shared by both A and B divided by the lowest number of taxa of A or B, multiplied by 100%.

The dominant families in the floras of southern and northwestern Yunnan are different, with the exception of the most dominant families (see Table 1). The families Zingiberaceae, Cucurbitaceae, Apocynaceae, Annonaceae, Convolvulaceae, Arecaceae and Sterculiaceae are dominant families in the flora of southern Yunnan, while Gentianaceae, Saxifragaceae, Salicaceae, Caryophyllaceae, Cruciferae, Papaveraceae, Campanulaceae, Berberidaceae and Crassulaceae are the dominant families in the flora of northwestern Yunnan.

A majority of the thirty most dominant genera in central Yunnan (Table 2) are also common to either southern or northwestern Yunnan. However, southern and northwestern Yunnan do not share any dominant genera.

Comparisons of geographical elements (distribution types) at family level from these regional floras revealed that, excluding cosmopolitan families, families with tropical distributions in all (Table 3, type 2–7) contribute to a majority of the total number of families in all three floras of Yunnan, and families with pantropic distributions make up the highest proportion among geographical elements.

Tropical genera in all (Type 2–7) make up 77.02% of the total number of genera in the flora of southern Yunnan, and genera with temperate distributions (Type 8–14) contribute only to 17.02% of the total genera, while in northwestern Yunnan tropical genera contribute to 42.87% and genera with temperate distributions contribute to 47.19% of the total genera. The flora of central Yunnan is comprised mainly of tropical genera (64.37%) and temperate genera contribute to 32.33%. Genera with tropical Asian and tropical Asia to tropical Australia distributions constitute a noticeably high proportion in the flora of southern Yunnan. Genera with north temperate, east Asia and north America disjunct, old world temperate, east Asia, and Chinese endemic distributions make up the highest proportion of flora in northwestern Yunnan.

Within these three regions, 352 genera are found only in southern Yunnan, while 375 genera are found only in northwestern Yunnan (Table 6). Among the genera only in southern Yunnan, 330 genera have tropical distributions, including 169 tropical Asian genera, 59 genera with pantropic distributions, and 44 genera with tropical Asia to tropical Australia distributions. Among the genera that are found only in northwestern Yunnan, 305 genera have a temperate distribution, including 78 genera from east Asia, 75 genera with north temperate distributions, 51 genera with old world temperate distributions and 39 Chinese endemic genera.

**Table 6:** Ggeographical elements of seed plants at the generic level in these floras respectively and shared by two of them.

Geographical elementsat generic level	Genera only in southern Yunnan				Genera only in northwestern Yunnan				Genera only in central and southern Yunnan				Genera only in central and northwestern Yunnan				
	No. of genus		%		No. of genus		%		No. of genus		%		No. of genus		%		
Cosmopolitan		4		1.14		12		3.20		7		1.13		22		3.31	
Pantropic		59		16.76		17		4.53		102		16.43		41		6.17	
Tropical Asia and Tropical America disjunct		6		1.70		4		1.07		18		2.90		15		2.26	
Old World Tropic		29		8.24		5		1.33		58		9.34		15		2.26	
Tropical Asia to Tropical Australia		44		12.50		4		1.07		78		12.56		10		1.51	
Tropical Asia to Tropical Africa		23		6.53		4		1.07		42		6.76		17		2.56	
Tropical Asia		169		48.01		24		6.40		253		40.74		49		7.38	
North Temperate		0		0.00		75		20.00		5		0.81		146		21.99	
East Asia and North America disjunct		3		0.85		31		8.27		10		1.61		48		7.23	
Old World Temperate		2		0.57		51		13.60		5		0.81		75		11.30	
Temperate Asia		0		0.00		10		2.67		1		0.16		13		1.96	
Mediterranean, W Asia to C Asia		0		0.00		10		2.67		0		0.00		12		1.81	
Center Asia		1		0.28		11		2.93		2		0.32		13		1.96	
East Asia		8		2.27		78		20.80		27		4.35		137		20.63	
Endemic to China		4		1.14		39		10.40		13		2.09		51		7.68	
Total no. of genera		352		100.00		375		100.00		621		100.00		664		100.00	

Of the genera that are shared by two of the three floras, 621 genera are found only in central and southern Yunnan, and 664 genera are present only in the floras of central and northwestern Yunnan. Among the genera found only in central and southern Yunnan, 551 genera have tropical distributions, including 253 genera from tropical Asia, 102 genera with pantropic distributions, and 78 genera with tropical Asia to tropical Australia distributions. Among the genera only to the floras of central and northwestern Yunnan, 495 genera have temperate distributions, including 146 north temperate genera, 137 genera of east Asia, and 75 genera with old world temperate distributions. It is evident that the flora of central Yunnan has floristic attributes of both southern and northwestern Yunnan, and is related to the flora of southern Yunnan mainly by tropical elements, especially tropical Asia and pantropic elements, while related to the flora of northwestern Yunnan mainly through temperate elements, especially north temperate and east Asian elements.

## Discussion and Conclusions

The flora of Yunnan is noticeably divergent along altitude, latitude and topography. Southern Yunnan has a tropical flora of Malaysian affinity, while northwestern Yunnan has a temperate Himalayan flora. Whether these floral patterns are a consequence of mainly ecological divergence or historical-biogeography is of important significance in revealing the formation and evolution of the flora of Yunnan.

Although the floras of southern and northwestern Yunnan have a similar composition at the family level (88% similarity), they differ at the generic (55.89% similarity) and especially at specific (26.72% similarity) levels.

The flora of northwestern Yunnan is dominated by families and genera with cosmopolitan and north temperate distributions, while the flora of southern Yunnan, although sharing some large cosmopolitan families, is additionally dominated by tropical families and genera.

The flora of northwestern Yunnan is comprised more of tropical families (45.18) than temperate families (24.7%), but temperate genera are more dominant (47.19%). The flora of southern Yunnan is comprised mainly of tropical families (57.92%) and genera (77.02%). Furthermore, the flora of northwestern Yunnan is characterized by a relatively high proportion of genera with northern temperate, east Asian and old world temperate distributions, while the flora of southern Yunnan is characterized by a relatively high proportion of genera with tropical Asian (making up the highest proportion), pantropic and tropical Asia to tropical Australia distributions. Among genera that are known from southern Yunnan but not central or northwestern Yunnan, the majority have tropical distributions. Among genera in northwestern Yunnan only, the majority have temperate distributions. These factors reveal that the flora of northwestern Yunnan is different from the flora of southern Yunnan in floristic attributes. The flora of northwestern Yunnan is temperate in nature, while the flora of southern Yunnan is tropical in nature and has strong tropical Asian affinities.

Furthermore, if we look at global species numbers from each family and consider the proportion of species that are found in each of these regional floras (excluding cosmopolitan families), the families characteristic of southern Yunnan are quite different from those of northwestern Yunnan. In the flora of southern Yunnan, tropical families, such as Tetramelaceae, Cardiopteridaceae, Sphenocleaceae, Sladeniaceae, Musaceae, Hernandiaceae, Gnetaceae and Opiliaceae are most characteristic; while in northwestern Yunnan, temperate families, especially those with east Asian distributions, such as Helwingiaceae, Tetracentraceae, Stachyuraceae, Cephalotaxaceae, Eupteleaceae, and those with north temperate distributions, such as Diapensiaceae, Caprifoliaceae, Hydrangeaceae, Betulaceae, Pinaceae, Juncaceae and Papaveraceae are characteristic.

The conspicuous divergence between the floras of southern and northwestern Yunnan might be attributed to differences in geological history as well as differences in ecological habitat. The northwestern Yunnan had a temperate and subtropical flora during the Tertiary [26], and underwent a quick uplift with the Himalayas after the Pleistocene [27]. Uplift of Himalaya mountains began about 50 Myr ago, and further significant increases in altitude of the Tibetan plateau are thought to have occurred about 10±8 Myr ago [28], [29], [30]. With the uplift of Himalaya, east Asian monsoons were appeared about 9-8 Myr ago, and intensified about 3.6–2.6 Myr ago [31]. Studies on fossil mammalian, pollen, as semblages and sedimentclay mineralogy as well as carbon isotope data from fossil tooth enamels and paleosol carbonates revealed that the central Himalaya had a much warmer environment in the late Neogene, and the “paleo-thermometer” were 21±6°C at c. 7 Myr ago, which is 19±6°C higher than the present-day, and it was suggested that these area have been raised by c.2–2.5 km since 7 Myr to its current elevation of 4100–4500 m above sea level [32]. The northwestern Yunnan, which is part of eastern Himalaya, should have the similar environmental changes and tectonic uplift as the central Himalaya since the late Neogene. On the other hand, collision between India and Asia also caused lateral extrusion of southeast Asia between 32 Ma and 10 Ma [28], [33], [34], [35], [36], [37].

It could be inferred that these floras of Yunnan could derived from a common Tertiary tropical or subtropical Asian flora. Thereafter, the flora of northwestern Yunnan has evolved with the uplift of the Himalayas by gradual proliferation of mainly cosmopolitan and north temperate floristic elements, while the flora of southern Yunnan has evolved with extrusion of the Indochina block to southeast Asia by the influence of mainly tropical Asian elements.
